# Multimodal Optimal Base Station Selection Network for Intelligent Communications

**DOI:** 10.3390/s25226895

**Published:** 2025-11-12

**Authors:** Haie Dou, Xinyu Zhan, Xinyu Zhang, Taojie Zhu, Lei Wang

**Affiliations:** 1 School of Communication and Information Engineering, Nanjing University of Posts and Telecommunications, Nanjing 210003, China; 2 Jiangsu Engineering Research Center of Communication and Network Technology, Nanjing University of Posts and Telecommunications, Nanjing 210003, China; 3 School of Digital Media and Design Art, Nanjing University of Posts and Telecommunications, Nanjing 210003, China

**Keywords:** multimodal information fusion, base station selection, channel state prediction, intelligent wireless networks

## Abstract

With the rapid development of next-generation wireless communication systems, the increasing density of heterogeneous base stations and the dynamic nature of channel conditions have posed significant challenges to accurate and timely base station selection. Traditional single-modal approaches relying solely on partial channel or location information often fail to capture the complex semantics of real communication scenarios, leading to suboptimal decision-making. To address these limitations, this paper proposes the Multimodal Optimal Base Station Selection Network (MOBS-Net), which integrates multimodal spatial and temporal information to achieve both optimal base station judgment and proactive prediction. The judgment module employs convolutional neural networks to extract image semantics and a Transformer-based fusion mechanism to combine image, location, and channel features for real-time decision-making. The prediction module leverages multimodal sequential data and a large-scale multimodal model to extract temporal semantics, enabling proactive base station switching under dynamic channel conditions. Extensive experiments demonstrate that MOBS-Net significantly outperforms single-modal baselines, achieving an accuracy of 92.20% for optimal base station judgment and 91.5% for prediction tasks. These results highlight the reliability and effectiveness of MOBS-Net for intelligent base station decision-making in dynamic wireless environments.

## 1. Introduction

With the rapid evolution of wireless communication technologies, sixth-generation (6G) networks are envisioned to deliver unprecedented data rates and ultra-low latency by exploiting abundant spectrum resources in high-frequency bands [1,2,3]. These high-frequency transmissions, typically operating in terahertz (THz) ranges and millimeter-wave (mmWave), offer substantial bandwidth that can theoretically support extremely high throughput and massive connectivity for ultra-dense heterogeneous deployments [4]. However, such promising characteristics are accompanied by considerable challenges, including aggravated path loss, limited transmission range, and high sensitivity to physical blockages [5,6,7].

Due to the critical dependence of high-frequency communications on LOS propagation, sudden obstructions can critically degrade link quality and network reliability [8,9]. In non-line-of-sight (NLOS) conditions, user equipment (UE) must be handed over seamlessly to alternative LOS base stations to maintain quality of service (QoS) [10]. Traditional base station selection mechanisms rely heavily on full channel state information (CSI) estimation across all candidate base stations. This approach not only introduces substantial pilot and feedback overhead in large-scale multiple-input multiple-output (MIMO) systems but also fails to enable proactive handovers before link blockage occurs. As a result, conventional channel prediction and base station selection methods struggle to cope with the highly dynamic and complex wireless environments of future 6G networks [11,12].

The integration of computer vision with wireless communication has recently attracted considerable research interest for facilitating the development of intelligent, context-aware 6G networks. Vision-aided communication frameworks leverage multi-modal perception to enhance situational awareness and address challenges such as dynamic blockage, beam management, and proactive base station (BS) handoff in millimeter-wave (mmWave) and THz systems. For instance, Ref. [13] integrates image and beam information to achieve blockage prediction and BS handoff. Although effective, this approach relies heavily on synthetic datasets and is limited to scenarios involving only two BSs. Similarly, Ref. [14] introduces a GRU-based model incorporating object detection for link-state prediction. However, it does not consider multi-BS cooperation, and its prediction window remains fixed, limiting adaptability under dynamic conditions. In addition, Ref. [15] combines GPS and visual information to perform beam prediction and validates its effectiveness using real-world data, but it does not address NLOS scenarios or BS switching mechanisms. Likewise, Ref. [16] proposes a two-stage vision-based blockage prediction framework based on real datasets, yet it lacks channel feature fusion and does not include beam optimization strategies. More recently, Ref. [17] exploits multi-camera inputs for BS selection and beam switching. Despite its innovation in multi-view fusion, it still depends on fixed-scene deployment and suffers from limited robustness under high-speed mobility.

Beyond visual-assisted frameworks, channel state prediction has emerged as another critical research topic for enabling intelligent BS selection and proactive handover in highly dynamic wireless environments. Depending on the temporal, frequency, and spatial correlations between known and unknown CSI, existing prediction approaches can be categorized into several directions, among which time-domain prediction constitutes one of the most important research focuses [18]. In [19], a Sliding Bidirectional Gated Recurrent Unit (SBGRU) was employed to construct an estimator for learning time-varying Rayleigh fading channels. By integrating a recurrent neural network (RNN) structure with a sliding-window mechanism, their model processes arbitrarily long transmission symbols and outputs predictions in real time [20]. Extensive simulations demonstrated that the SBGRU estimator outperforms traditional algorithms and other neural networks in dynamically tracking channels, exhibiting strong robustness under different pilot densities. Similarly, Ref. [21] developed an end-to-end system that integrates long short-term memory (LSTM) and residual networks (ResNet) to process channel sequences. By learning from historical data, their approach captures intrinsic temporal relationships and achieves accurate future CSI prediction for link scheduling optimization [22,23].

Another active line of research is frequency-domain extrapolation, which estimates unknown CSI at unmeasured frequencies using known CSI, thereby reducing pilot overhead. Depending on the prediction range, this can be further divided into intra-band and cross-band extrapolation. For intra-band estimation, Ref. [24] introduced a deep learning-based joint channel estimation and signal detection framework for OFDM systems. Exploiting time–frequency correlation, they designed a Channel Estimation Network (CENet) and a Channel Conditioned Recovery Network (CCRNet), both of which outperform traditional methods and exhibit high robustness. Building upon this, Ref. [25] proposed a deep learning-based approach to infer unknown channel parameters from a small subset of known parameters by approximating the mapping between parameter subspaces. The authors analyzed three extrapolation scenarios—antenna, frequency, and terminal domains—highlighting challenges in reconfigurable intelligent surface (RIS) systems [26,27] and large-scale MIMO.

Another popular direction involves predicting downlink CSI from uplink CSI, which varies depending on the duplexing scheme [28,29]. In time division duplexing (TDD) systems, the problem reduces to temporal extrapolation due to channel reciprocity, whereas in frequency division duplexing (FDD) systems, it becomes a joint time–frequency extrapolation task due to non-overlapping frequency bands. To address these challenges, Ref. [30] proposed a data-driven CNN–GAN framework to infer downlink CSI from uplink CSI by leveraging the shared propagation environment. A boundary-equilibrated GAN with MSE loss was adopted to improve convergence and capture the joint distribution of CSI. Similarly, Ref. [31] utilized a deep neural network (DNN) to model intrinsic uplink–downlink correlations, incorporating probabilistic antenna subset selection to optimize channel extrapolation and reduce data dimensionality. Their results demonstrated effective large-scale MIMO channel prediction with strong robustness.

Overall, while vision-aided communication studies have demonstrated the feasibility of integrating visual perception for proactive blockage prediction, and channel prediction research has made substantial progress in CSI inference across domains, both lines of work still face key limitations. Existing vision-assisted methods often rely on constrained datasets and lack multi-BS scalability, whereas conventional channel prediction frameworks are limited by unimodal inputs and insufficient adaptation to real-world dynamics. These limitations drive the need for a unified multimodal framework that integrates visual context, channel features, and spatial–temporal correlations to achieve robust blockage prediction and adaptive beam management in next-generation 6G communication systems.

Despite these advances, most state-of-the-art channel prediction algorithms focus solely on CSI-based sensing and lack the integration of environmental information, which may be insufficient to support the complex and dynamic scenarios envisioned for future 6G communications [32]. Motivated by this gap, this paper explores the fusion of multimodal information, including channel states and environmental perception, to enhance the sensing of channel dynamics and enable more accurate and proactive base station selection. Specifically, we propose a Multimodal Optimal Base Station Selection Network (MOBS-Net) that integrates heterogeneous features from the wireless channel and surrounding environment to achieve timely and reliable base station switching under dynamic high-frequency scenarios.

Different from existing multimodal fusion approaches such as ViT-based models, which primarily focus on global attention over image patches [33], and cross-attention mechanisms that emphasize feature alignment between modalities [34], MOBS-Net introduces a communication-driven multimodal fusion framework tailored for dynamic base station selection. Moreover, vision–language models such as CLIP [35] and BLIP-2 [36] demonstrate strong capability in semantic alignment across modalities, yet they are designed mainly for perception tasks and lack optimization for wireless communication objectives. In contrast, MOBS-Net addresses the limitations of conventional methods by proposing a hierarchical semantic fusion mechanism that integrates local visual details (extracted via CNNs to capture obstacles and base station geometry), channel state information (mapped to spectral features via Fourier transform to model beamforming vectors and signal-to-noise ratio), and spatial coordinates (encoded with user trajectory and base station deployment density as semantic anchors). Furthermore, MOBS-Net innovates through a dual-network collaborative architecture, where a judgment network first filters candidate base stations via sigmoid-based binary classification to reduce redundant modal inputs, and a prediction network then leverages a multimodal large model to forecast long-term performance metrics (e.g., handover latency and throughput) using cross-entropy loss optimization, thereby balancing real-time decision-making and prediction accuracy in high-mobility scenarios. To accommodate edge computing limitations in base stations, a lightweight modal adaptation module is also introduced, compressing visual and channel features via 1 × 1 convolutions and spectral mapping to reduce parameter size by 80% compared to traditional ViT models while maintaining inference speed suitable for dynamic communication environments. This paper makes several key contributions, which are summarized as follows:

- Multimodal Optimal Base Station Selection Network. We propose MOBS-Net, which combines two complementary modules: a real-time base station judgment network and a sequential base station prediction network. This unified framework enables both accurate current-time base station selection and proactive prediction of the optimal base station at future time slots.
- Real-time multimodal judgment network. The judgment module utilizes a convolutional neural network for image feature extraction and a Transformer-based mechanism for multimodal fusion. By integrating channel state, user location, and environmental perception data, this approach enhances the accuracy and robustness of optimal base station selection.
- Sequential multimodal prediction network. The prediction module exploits multimodal temporal data and introduces a large-scale multimodal model to capture sequential semantics and refine task-specific predictions. This design enhances the continuity of environment understanding and enables preemptive base station switching before LOS link blockage occurs. Experimental results demonstrate that our framework achieves 92.20% accuracy for base station judgment and 91.5% for prediction, significantly outperforming single-modal approaches.

This paper proceeds as follows. Section 2 is dedicated to establishing the system model and formulating the problem. Section 3 details the proposed Multimodal Optimal Base Station Selection Network (MOBS-Net) and its key components. Section 4 evaluates the framework’s performance under the defined experimental setup. The concluding Section 5 is devoted to summarizing the research and proposing avenues for further investigation.

## 2. High-Frequency Wireless Communication System Modeling

### 2.1. System Model

**Figure 1:**
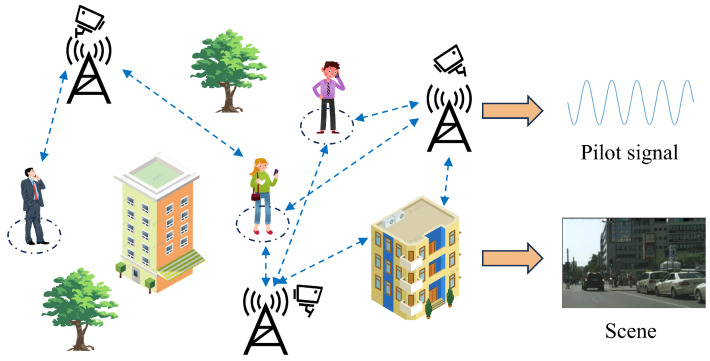
Multi-base station multi-user communication scenario.

As depicted in Figure 1, this chapter investigates an outdoor communication scenario with multiple users and small base stations. The scenario consists of three small base stations, each configured with a single radio-frequency (RF) chain and a uniform linear array (ULA) of $N_{t}$ antennas. In addition, each small base station is outfitted with an RGB camera capable of capturing a sequence of images in a given direction. These images include the users currently served by the base station.

Assume that the base stations provide services to $N_{u}$ users, each equipped with $N_{r}$ antennas. During signal transmission, each base station performs beamforming using a predefined beamforming codebook. The codebook of base station *b* is denoted as

(1) $$F_{b}=\left\{f_{1},f_{2},\ldots ,f_{M_{b}}\right\},$$

where $b\in N_{b}=\left\{1,\;2,\;3\right\}$, $N_{r}$ denotes the antenna count in the ULA array of each base station and $M_{b}$ denotes the size of the beamforming codebook.

Since the beamforming vectors are determined by the selected antenna array, each beamforming vector in the codebook takes the form:

(2) $$f_{m}=\frac{1}{\sqrt{N_{t}}}{\left[\begin{array}{c} 1,e^{j\frac{2\pi }{\lambda }d\;\sin\left(\phi_{m}\right)},\ldots ,e^{j\left(N_{t}-1\right)\frac{2\pi }{\lambda }d\;\cos\left(\phi_{m}\right)} \end{array}\right]}^{T},$$

where λ denotes the wavelength, *d* defines the spacing between array elements, and $\phi_{m}=\frac{2\pi m}{M}$ is the azimuth angle with uniform quantization, where the step size is determined by $m\in M_{b}$.

The communication system in this work adopts an orthogonal frequency-division multiplexing (OFDM) framework comprising *K* subcarriers. Assume that, at time slot *t*, base station *b* serves user *u*. The downlink signal at user *u* is given by

(3) $$y_{u,k}=h_{k}^{T}f_{m}x_{b,k}+n_{k},$$

where $y_{u,k}\in C$ is the received signal at user *u* on the *k*-th subcarrier, $h_{k}\in C^{N_{t}\times N_{r}}$ represents the channel matrix between base station *b* and user *u*, and $x_{b,k}\in C$ denotes the transmitted signal from base station *b* to user *u*, satisfying the power constraint $E[|x_{b,k}{|}^{2}]=P$ with *P* being the per-symbol power budget. The noise term $n_{k}$ follows a complex Gaussian distribution $N_{C}\left(0,\sigma^{2}\right)$.

Accordingly, the transmission rate achieved by base station *b* for user *u* can be written as

(4) $$R_{u,t}=B_{u,t}\log_{2}\;\left(1+\frac{{\left|h_{k}^{T}f_{m}\right|}^{2}}{\sigma_{n}^{2}}\right),$$

where $B_{u,t}$ denotes the channel bandwidth allocated to user *u* at time slot *t*. In high-frequency communication systems, to maximize the downlink transmission rate, users should preferentially select the base station under a LOS link.

### 2.2. Channel Model

This chapter utilizes a geometric millimeter-wave channel model. The channel state information of user *u* on the *k*th subcarrier is expressed by

(5) $$h_{k}=\sum_{d=0}^{D}\sum_{l=1}^{L}\alpha_{l}e^{-j\frac{2\pi k}{K}d}p\left(dT_{S}-\tau_{l}\right)a\left(\theta_{l},\phi_{l}\right),$$

where *D* is the cyclic prefix length in the OFDM system, *L* denotes the number of channel paths, $\alpha_{l}$ and $\tau_{l}$ represent the gain (encompassing path loss) and delay of the *l*-th path respectively, $\theta_{l}$ and $\phi_{l}$ are the respective azimuth and elevation angles, and $T_{S}$ is the sampling interval.

Equation (5) provides a general description of whether the channel between the user and the base station is LOS or NLOS. To explicitly distinguish between LOS and NLOS channels, we further modify the expression. Let $w={\left[w_{1},\ldots ,w_{L}\right]}^{T}$, where for all l∈{1,…,L}, $w_{l}\in \left\{0,1\right\}$, and w is a binary indicator vector. Without loss of generality, the first path in Equation (5) is defined as the direct LOS path from the user to the base station, i.e., l=1. Consequently, the geometric channel model in (5) can be expressed as

(6) $$h_{k}=\sum_{d=0}^{D}\sum_{l=1}^{L}w_{l}^{u}\;\left[\alpha_{l}e^{-j\frac{2\pi k}{K}d}p\left(dT_{S}-\tau_{l}\right)a\left(\theta_{l},\phi_{l}\right)\right],$$

where, specifically, if the binary vector w associated with user *u* satisfies $w_{1}^{u}=0$, the user is considered to be in an NLOS state; conversely, if $w_{1}^{u}=1$, the user is regarded as being in an LOS state.

### 2.3. Problem Formulation

In the absence of obstacles, the link between a base station and a user in a high-frequency communication system operates in a LOS state. In this case, the nearest base station to the user typically provides the strongest signal power and thus serves as the optimal communication base station. However, the appearance of obstacles between the base station and the user causes a rapid deterioration in the user’s received signal power, potentially resulting in communication interruptions. Under such conditions, the user–base station link becomes NLOS, and a base station handover is required. Therefore, how to select the optimal base station for continuously moving users in a dense base station scenario becomes an urgent problem. In this chapter, the problem is divided into two subproblems: optimal base station selection at the current time and optimal base station prediction at future times, which are referred to as the optimal base station identification problem and the optimal base station prediction problem, respectively.

#### 2.3.1. Optimal Base Station Identification Problem

To address the optimal base station selection problem, the key lies in enabling the wireless network to perceive the surrounding environment. Such perceptive capability allows the network to make accurate decisions in complex environments. Specifically, the network should be able to determine the optimal communication base station for a user at a given time based on the scene information. Therefore, this chapter proposes to employ a NDD to fuse image information with partial CSI to identify the optimal communication base station for a user at time *t*. The rationale for choosing these two modalities is as follows: (1) In general, visual data contains rich information about the depicted scene, such as user positions and obstacle locations; video data additionally provides information about user movement. The features extracted from multi-view images are referred to in this chapter as image semantics. (2) The parameters constituting the channel matrix are influenced by both the scene’s geometry and its electromagnetic properties. While geometric features are extractable from image data, electromagnetic properties depend on multiple factors, including building materials, vehicle surfaces, and weather conditions. To enhance the perception of the electromagnetic environment, this chapter incorporates a portion of the user’s channel matrix as an additional modality. The features derived from this partial matrix are designated as channel semantics.

The image information is captured via cameras mounted above the base stations. Let the multi-view images collected at time *t* be denoted by

(7) $$\{\left(I_{1}\left[t\right],I_{2}\left[t\right],I_{3}\left[t\right]\right)\},$$

where $I_{b}\left[t\right]\in R^{W\times H\times C}$ is the single-view image captured by base station *b* at time *t*. The partial channel matrix, denoted by $C_{u}\left[t\right]$, can be obtained through pilot transmission and channel estimation on a subset of antennas.

Another critical issue is that multiple users exist in the scene, necessitating an additional modality to identify each user. Since user positions do not overlap at the same time, this chapter introduces user position information to assist the network in recognizing the target user, denoted by $G_{u}\left[t\right]$.

Thus, the multi-view image information, user position information, and partial channel matrix information are jointly referred to as the scene information, which can be expressed as

(8) $$S_{u}\left[t\right]=\left\{\left(I_{1}\left[t\right],I_{2}\left[t\right],I_{3}\left[t\right]\right),G_{u}\left[t\right],C_{u}\left[t\right]\right\}.$$

Based on the scene information $S_{u}\left[t\right]$, the optimal base station for user *u* at time *t* can be determined through a mapping defined as

(9) $$\Gamma_{1}:S_{u}\left[t\right]\to b^{*},$$

where $b^{*}$ denotes the optimal base station for user *u* at time *t*. Since an explicit mathematical function for the mapping Equation (9) is difficult to obtain, this chapter employs a DNN to approximate $\Gamma_{1}$ with the aid of training datasets. The optimal base station identification function:

(10) $$b^{*}=f\;\left(S_{u}\left[t\right],\Psi \right),$$

where Ψ denotes the parameters of the DNN.

#### 2.3.2. Optimal Base Station Prediction Problem

Although the DNN proposed in the previous subsection can determine the optimal serving base station of a user at a given time instant, this approach is inherently reactive. For example, if the optimal base station inferred from the current scene information is different from the one to which the user is currently connected, a handover procedure must be initiated after the optimal base station has been identified. During this period, the communication link of the user may already be blocked. Consequently, relying solely on the current scene information cannot proactively support users in selecting the optimal base station before a blockage occurs; it can only enable reactive handovers after the blockage has happened.

Enabling low latency handoffs in high-frequency wireless systems, it is essential to endow the network with the capability of continuously perceiving the surrounding environment. Such perception enables the network to shift from passive responses to proactive adaptation. Specifically, the network should be able to predict in advance the optimal base station for a user at a future time instant and trigger the handover process proactively. Therefore, this subsection attempts to predict the future optimal base station of a user by leveraging multi-view image information and partial channel state information collected over a past time window.

In real-world scenarios, a user’s state does not change abruptly. Hence, the future scene is predictable via the analysis of states over the past *T* time instants, and this mapping relationship may be formalized as

(11) $$\Gamma_{2}:\left\{S_{u}\left[t-T\right],S_{u}\left[t-T+1\right],\cdots S_{u}\left[t-1\right]\right\}\to S_{u}\left[t\right],$$

where $\left\{S_{u}\left[t-T\right],S_{u}\left[t-T+1\right],\cdots S_{u}\left[t-1\right]\right\}$ denotes the multi-modal scene information set over the past *T* time instants.

Since Equation (9) has already established that the optimal base station at time *t* can be determined from $S_{u}\left[t\right]$, combining these two mappings yields:

(12) $$\Gamma_{3}:\left\{S_{u}\left[t-T\right],S_{u}\left[t-T+1\right],\cdots S_{u}\left[t-1\right]\right\}\to b^{*}.$$

Therefore, the optimal base station at time *t* can be predicted based on the multi-modal scene information over the past *T* time instants.

Since the mapping in Equation (10) cannot be easily expressed in a closed-form mathematical function, we similarly employ a DNN to approximate $\Gamma_{3}$. The prediction function can be expressed as

(13) $$b^{*}=f^{\prime }\left(S_{u}\left[t\right],\Psi^{\prime }\right),$$

where $\Psi^{\prime }$ denotes the parameters of the DNN.

## 3. Multimodal Optimal Base Station Selection Network

To address the two problems identified in the previous subsection, this section proposes the design of a Multimodal Optimal Base Station Selection Network (MOBS-Net). The architectures of these networks are described separately below.

### 3.1. Optimal Base Station Judgment Network

**Figure 2:**
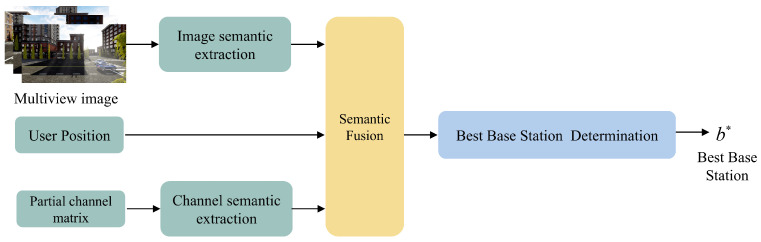
Best base station judgment network.

This subsection presents an Optimal Base Station Judgment Network based on multimodal scene information to assist users in selecting the optimal communication base station at time *t*, as illustrated in Figure 2. By fusing features from different information sources, richer scene semantics are extracted to improve the accuracy of determining the optimal base station for user *u* at time *t*.

Feature extraction is first conducted on each modality of the raw data. For the visual modality, multi-view images are initially transformed by an image encoder to mitigate their inherent redundancy. The image encoder utilizes a set of CNNs, which are widely adopted in image processing. Each CNN architecture comprises a series of convolutional layers culminating in a linear output layer, where the transformation at each convolutional stage is mathematically represented by

(14) CNNCell(x)=MaxPool(ReLU(BatchNorm(Conv(x)))),

where Conv(·) denotes the convolutional layer function, BatchNorm(·) normalizes the layer input by re-centering and re-scaling, ReLU(·) is a nonlinear activation function, and MaxPool(·) is a discretization function that downsamples the input representation.

The mathematical operation of the linear layer is formulated as

(15) $$\omega \left(x\right)=\mathrm{ReLU}\left(W_{c}*x+b_{c}\right),$$

where $W_{c}$ is the weight matrix of the linear layer and $b_{c}$ is the corresponding bias vector. Consequently, the output of the image encoder can be expressed as

(16) $$E_{b}^{I}\left[t\right]=\omega \left({\mathrm{CNNCell}}_{n}\left(\cdots \mathrm{CNNCell}\left(I_{b}\left[t\right]\right)\right)\right),$$

where $E_{b}^{I}\left[t\right]$ represents the image semantic information extracted from $I_{b}\left[t\right]$.

The user’s position is represented as a vector through a fully connected layer, denoted as the positional semantic $E^{G}\left[t\right]$. For the partial channel matrix $C_{u}\left[t\right]$, which is a downsampled representation of the channel information, the extracted channel semantic is denoted as $E^{C}\left[t\right]$.

These three types of semantic information are concatenated into a high-dimensional vector to form the preliminary scene semantics, which can be expressed as

(17) $$E\left[t\right]=\left\{\left\{E_{b}^{I}\left[t\right]\right\},E^{G}\left[t\right],E^{C}\left[t\right]\right\}.$$

### 3.2. Multimodal Semantic Fusion and Optimal Base Station Classification Network

To further integrate multimodal scene semantics, the semantic fusion module employs a multi-head Transformer encoder to map E[t] into a unified semantic space, leveraging the self-attention mechanism, the encoder extracts cross-modal dependencies and outputs high-level scene semantics that contain multimodal correlations. This process can be expressed as

(18) $$E_{r}=\mathrm{Transformer}\left(E[t]\right),$$

where $E_{r}$ denotes the deeply fused scene semantics considering multimodal information.

Based on $E_{r}$, an optimal base station classification network is designed. This network contains $L_{b}$ fully connected layers, whose output is formulated as

(19) $$Y_{r}=\mathrm{FCNN}\left(E_{r}\right)=\varphi^{L_{b}}\left(\cdots \varphi^{1}\left(E_{r}\right)\right),$$

where $Y_{r}={\left[y_{1},y_{2},\cdots ,y_{n_{r}}\right]}^{T}$, $n_{r}$ is the number of neurons in the last fully connected layer, and φ(·) denotes the mapping function of each fully connected layer.

Given that optimal base station selection is a multi-class classification task, the classification network employs a Softmax output layer to generate a probability distribution over all base stations, which is expressed as

(20) $$p_{b}=\mathrm{Softmax}\left(Y_{r}\right)=\frac{\exp(y_{b})}{\sum_{j=1}^{n_{r}}\exp\left(y_{j}\right)},$$

where $p_{b}$ represents the probability that base station *b* is the optimal one.

The loss function corresponding to base station classification may be formulated with cross-entropy loss:

(21) $${\mathrm{Loss}}_{r}=-\sum_{b=1}^{3}q_{b}\log\left(p_{b}\right),$$

where $q_{b}={\left[q_{1},q_{2},q_{3}\right]}^{T}$ is the label vector of the optimal base station classification task in a one-hot format. If base station *b* is the optimal one, then $q_{b}=1$; otherwise, $q_{b}=0$.

### 3.3. Multimodal Large Model-Enabled Optimal Base Station Prediction Network

**Figure 3:**
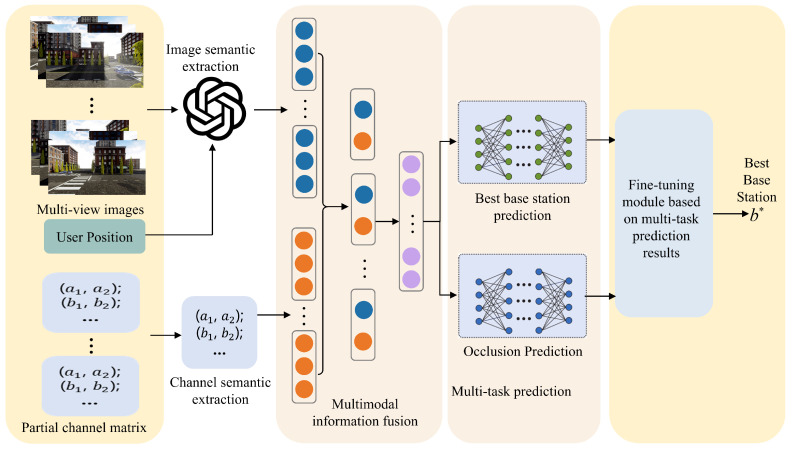
Optimal base station prediction network enabled by multimodal large models, $b^{*}$ represents the best base station.

This subsection proposes a multimodal large-model-enabled optimal base station prediction network to assist users in predicting the optimal communication base station at time *t*, as illustrated in Figure 3. By extracting features from temporal scene information, the network can predict the variation of channel states and thus estimate the likelihood of the optimal base station for the user at time *t*.

First, the sequential information of each modality is processed for feature extraction. For the multi-view image sequences captured by multiple base stations, where each sequence contains rich visual semantics as well as a large amount of redundant information, traditional convolutional networks are often insufficient to accurately track the same user across multiple scene images. In contrast, the proposed multimodal large model can leverage the initially provided user features to localize the target user within the scene and maintain user consistency across temporal variations.

Therefore, this subsection adopts a multimodal large model Ω (specifically implemented using the GPT-4 Vision model) combined with user features to extract semantic information from sequential images. The GPT-4 Vision model processes each image together with a textual prompt describing the target user (position, shape, color, etc.) to identify and locate the user and obstacles within the scene. The extracted visual embeddings serve as conceptual representations of multimodal perception, demonstrating the capability of large vision–language models to enhance spatial understanding without additional fine-tuning or re-training. The resulting sequential image semantics are denoted as

(22) $$F_{b}^{I}=\Omega \left(I_{b}\left[m\right],F^{u}\right),$$

where Ω(·) denotes the multimodal large model used to extract image semantics from sequential images, $F^{u}$ represents the user features (including initial position, color, shape, etc.), and $I_{b}\left[m\right]$ (t−T≤m≤t−1) denotes the image input at time *m*. $F_{b}^{I}=\left\{f_{b}^{I}\left[t-T\right],f_{b}^{I}\left[t-T+1\right],\ldots ,f_{b}^{I}\left[t-1\right]\right\}$ is an image semantic vector sequence, where $f_{b}^{I}\left[m\right]$ represents the image semantic vector of the image captured by base station *b* at time *m*.

For the partial channel matrix sequence $\left\{C_{u}\left[t-T\right],C_{u}\left[t-T+1\right],\ldots ,C_{u}\left[t-1\right]\right\}$, a fully connected neural network is employed to extract channel information, which is denoted as a channel semantic vector. The channel semantic vectors over the past *T* time steps are denoted as $F_{u}^{C}=\left\{f_{u}^{C}\left[t-T\right],f_{u}^{C}\left[t-T+1\right],\ldots ,f_{u}^{C}\left[t-1\right]\right\}$, where $f_{u}^{C}\left[m\right]$ denotes the channel semantics of user *u* at time *m*.

Then, based on a Transformer encoder, a multimodal semantic information fusion module is designed, to fuse the sequential image semantics $F_{b}^{I}$ and the sequential channel semantics $F_{u}^{C}$, thereby enhancing cross-modal interactions and extracting higher-level temporal semantics. First, the image and channel semantics from time t−T to t−1 are concatenated into the primary scene semantic sequence $E^{IC}$, and then the Transformer encoder performs semantic fusion as follows:

(23) $$E_{r}^{\prime }=\mathrm{Transformer}\left(E^{IC}\right),$$

where $E_{r}^{\prime }=\left\{e^{\prime }\left[t-T\right],e^{\prime }\left[t-T+1\right],\ldots ,e^{\prime }\left[t-1\right]\right\}$ is the multimodal fused scene semantics over the entire sequence. The output at the last time step $e^{\prime }\left[t-1\right]$ is used for subsequent optimal base station prediction.

Blockage conditions between the user and base stations directly influence the prediction of the optimal base station. Therefore, accurately identifying such blockages substantially enhances selection performance. Accordingly, this subsection formulates the prediction of the blockage state between the user and each base station at time *t* as an auxiliary task to the main base station selection process, as illustrated in Figure 3. Here, optimal base station prediction and blockage prediction are denoted as Task 1 and Task 2, respectively.

For Task 1, the designed prediction network contains $L_{1}$ fully connected layers. Assuming that the last layer has $n_{1}$ neurons, its output can be written as

(24) $$Y_{1}={\mathrm{FCNN}}_{1}\left(E_{r}^{\prime }\right)=\varphi^{L_{1}}\left(\cdots \varphi^{1}\left(E_{r}^{\prime }\right)\right),$$

where $Y_{1}={\left[y_{11},y_{12},\ldots ,y_{1,n_{1}}\right]}^{T}$. Given that Task 1 involves a multiclass classification, its output layer employs the Softmax function:

(25) $$p_{1,b}=\mathrm{Softmax}\left(Y_{1}\right)=\frac{\exp(y_{1,b})}{\sum_{j=1}^{n_{1}}\exp\left(y_{1,j}\right)},$$

where $p_{1,b}$ denotes the probability that base station *b* is the optimal base station. The cross-entropy loss for Task 1 is

(26) $${\mathrm{Loss}}_{1}=-\sum_{b=1}^{3}q_{1,b}\log\left(p_{1,b}\right),$$

where $q_{1}={\left[q_{11},q_{12},q_{13}\right]}^{T}$ is the one-hot label vector.

Task 2 aims to predict the blockage state between the user and each base station at time *t*. Its prediction network consists of $L_{2}$ fully connected layers:

(27) $$Y_{2}={\mathrm{FCNN}}_{2}\left(E_{r}^{\prime }\right)=\varphi^{L_{2}}\left(\cdots \varphi^{1}\left(E_{r}^{\prime }\right)\right).$$

Because the blockage states are mutually independent, Task 2 is a multi-label classification problem. The output layer uses the Sigmoid function:

(28) $$p_{2,b}=\mathrm{Sigmoid}\left(Y_{2}\right)=\frac{1}{1+\exp(-y_{2,b})},$$

where $p_{2,b}\in \left(0,1\right)$ represents the probability that there is no blockage between the user and base station *b*. The label vector for Task 2 is $q_{2}={\left[q_{21},q_{22},q_{23}\right]}^{T}$, with each element equal to 0 or 1. If there is blockage between the user and base station *b*, then $q_{2,b}=0$; otherwise, $q_{2,b}=1$. The loss of Task 2 is given by the binary cross-entropy for each base station:

(29) $${\mathrm{Loss}}_{2}=-\sum_{b=1}^{3}\left[q_{2,b}\log\left(p_{2,b}\right)+\left(1-q_{2,b}\right)\log\left(1-p_{2,b}\right)\right].$$

**Figure 4:**
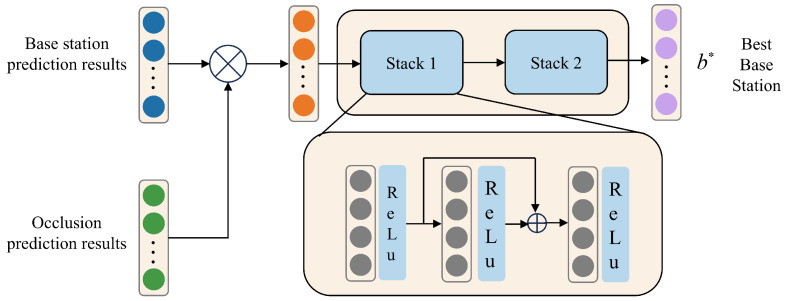
Fine-tuning the network, $b^{*}$ represents the optimal base station.

Considering the rapid attenuation and poor diffraction ability of high-frequency beams, if the link between base station and the user *b* is blocked, the received power decays exponentially, making *b* unlikely to be the optimal base station. Based on the predictions of Task 1 and Task 2, a fine-tuning network is designed, as shown in Figure 4, to further refine the prediction results. The input of the fine-tuning network is denoted as $p_{\mathrm{in}}=p_{2}⊙p_{1}$, where $p_{2}=\left[p_{2,1},p_{2,2},p_{2,3}\right]$ and $p_{1}=\left[p_{1,1},p_{1,2},p_{1,3}\right]$. The network contains two stacks of three fully connected layers each. Each layer connects to all previous layers, mitigating gradient vanishing and improving feature propagation. The *j*-th stack output is formulated as

(30) $$\begin{array}{cc} V_{j,1} & =\mathrm{ReLU}\left(W_{j,1}p_{f}+b_{j,1}\right),\\ V_{j,2} & =\mathrm{ReLU}\left(W_{j,2}\left(p_{f}+V_{j,1}\right)+b_{j,2}\right),\\ V_{j,3} & =\mathrm{ReLU}\left(W_{j,3}\left(p_{f}+V_{j,1}+V_{j,2}\right)+b_{j,3}\right), \end{array}$$

where $p_{f}$ is the input feature to the current stack.

After processing through two stacking layers and a Softmax layer, the fine-tuning network generates the final output representation:

(31) $$p_{\mathrm{out}}=\mathrm{Softmax}\;\left(ST\left(p_{\mathrm{in}}\right)\right),$$

where ST(·) represents the stacking operation and $p_{\mathrm{out}}=\left[p_{\mathrm{out},1},p_{\mathrm{out},2},p_{\mathrm{out},3}\right]$ corresponds to the output probability vector containing the refined selection probabilities for the optimal base station.

The prediction loss is computed using the cross-entropy function:

(32) $${\mathrm{Loss}}_{3}=-\sum_{b=1}^{3}q_{3,b}\log\left(p_{\mathrm{out},b}\right),$$

where $q_{3}$ denotes the label of the optimal base station, i.e., $q_{3}=q_{1}$.

By combining the losses from Task 1, Task 2, and the fine-tuning network, the total loss of the multimodal large-model-empowered base-station prediction network is given by

(33) $$\mathrm{Loss}=\lambda_{1}\;{\mathrm{Loss}}_{1}+\lambda_{2}\;{\mathrm{Loss}}_{2}+\lambda_{3}\;{\mathrm{Loss}}_{3},$$

where $\lambda_{1},\lambda_{2},\lambda_{3}\geq 0$ are the weighting coefficients of the three loss terms.

## 4. Simulation and Analysis

### 4.1. Simulation Setup

This section presents simulation experiments employing the first connected intelligent vehicle dataset developed by Peking University. The dataset integrates both wireless communication and multi-modal perception data, encompassing diverse communication scenarios. The dataset currently comprises 160,000 channel impulse response (CIR) matrices, 180,000 RGB images, 180,000 LiDAR point clouds, 360,000 depth images, and 100,000 radar signal waveforms.

The experiments are conducted using the M3SC dataset [37], which was developed by Peking University for connected intelligent vehicle research. The dataset covers outdoor urban intersection environments and includes synchronized RGB camera images, LiDAR point clouds, radar signals, and wireless channel impulse responses collected by roadside units and vehicle-mounted sensors. The dataset includes data collected at an intersection scenario. In this work, a subset under sunny conditions is used, which contains continuous image sequences, successive channel state matrices, and vehicle position information collected by three roadside units. The roadside units, referred to in this study as small millimeter-wave base stations, are renumbered as Base Station 1, Base Station 2, and Base Station 3, with their specific parameters listed in Table 1. The cameras are mounted 3.3 m above each base station. Subsequently, the best base station for each vehicle at each time step and the blockage status between the vehicle and each base station are determined to serve as labels for the subsequent training process.

It should be noted that the three base stations in this study are simulated within the M3SC experimental framework and are not deployed by any network operator. They emulate 5G-compatible millimeter-wave small cells operating under identical communication parameters and are controlled by a unified experimental platform. This simulation setup enables a fair evaluation of multimodal base station selection under reproducible conditions. In the simulation setup, line-of-sight (LOS) and non-line-of-sight (NLOS) conditions are modeled, where L = 1 corresponds to the LOS path. The M3SC dataset provides channel matrices generated through ray-tracing, which include multipath propagation effects under realistic outdoor vehicular scenarios.

To ensure scalability in large-scale network topologies, the proposed MOBS-Net employs a localized selection strategy. For each user, only the three nearest base stations are considered based on spatial proximity and signal strength, and the line-of-sight (LOS) links are prioritized for candidate filtering. This design allows the model to maintain low computational complexity and stable performance even in dense multi-cell deployments.

All experiments were implemented in Python 3.10 using the PyTorch 2.1 framework with CUDA 12.1 for GPU acceleration. The simulations were executed on a workstation equipped with an Intel Core i9-13900K CPU, an NVIDIA RTX 4090 GPU, 64 GB of RAM. The batch size was set to 32 for both training and inference.

### 4.2. Configuration of Base Station Networks

In the following, the configurations of the base station judgment network and base station prediction network are described.

#### 4.2.1. Base Station Judgment Network  Configuration

The images acquired by the cameras have a resolution of 1920 × 1080 pixels. A three-layer convolutional neural network (CNN) is designed for feature extraction. The first convolutional layer uses a 7 × 7 kernel, stride 2, padding 3, with 32 output channels, followed by a 2 × 2 max pooling layer, reducing the image size to 960 × 540. The second layer uses a 5 × 5 kernel, stride 2, 64 output channels, and further pooling reduces the size to 480 × 270. The third layer uses a 3 × 3 kernel, 128 output channels, reducing the size to 240 × 135. The resulting 3D feature tensor is flattened and passed through a fully connected layer to generate a 128-dimensional image semantic vector for subsequent multimodal fusion.

To enhance location information, the vehicle’s position is encoded into a 32-dimensional vector for use in multimodal fusion. Channel semantics are extracted from partial channel matrices via a two-layer perceptron: the first layer outputs 128 dimensions, the second outputs 64 dimensions, with ReLU activations for all layers.

The image, location, and channel semantic vectors are concatenated, embedded, and input into a Transformer encoder for fusion. The Transformer consists of two encoding layers, each with 8 attention heads of 32 dimensions (total 256), a feedforward hidden layer of size 256 with ReLU activation, residual connections, layer normalization, and a dropout of 0.1. To simplify parameters and improve efficiency, a linear mapping compresses the fused semantic vector from 256 to 128 dimensions.

The judgment network comprises four fully connected layers, with their dimensions set to 128, 64, 16, and 3 respectively. A Softmax output layer is also included to acquire the probabilities of base station selection. The model is optimized with the Adam algorithm, with its initial learning rate set at 0.001.

#### 4.2.2. Base Station Prediction Network  Configuration

For the base station prediction network, the sequence length is 4. GPT-4-vision-preview is used to extract image semantics from each sequence, compressed to a 128-dimensional image semantic vector via a fully connected layer. The model identifies the target vehicle and possible obstacles in the image sequence. Channel semantics are similarly compressed to 64 dimensions. Multimodal semantic vectors at each timestep are concatenated to form a sequence.

An embedded representation of the sequence is processed by a two-layer Transformer encoder. Each layer has 4 attention heads of 64 dimensions (total 256), a feedforward hidden layer of 256 with ReLU activation, residual connections, layer normalization, and 0.1 dropout. A linear mapping reduces the fused vector from 256 to 128 dimensions.

For the best base station prediction task, a four-layer fully connected network is used with layer dimensions 128, 64, 16, and 3, with a Softmax output. The blockage prediction task uses a similar four-layer network with Sigmoid output for 3 dimensions (one per base station). The fine-tuning network has 3 neurons per layer to output the final probability vector.

### 4.3. Simulation Results and Analysis

#### 4.3.1. Base Station Judgment Network

**Figure 5:**
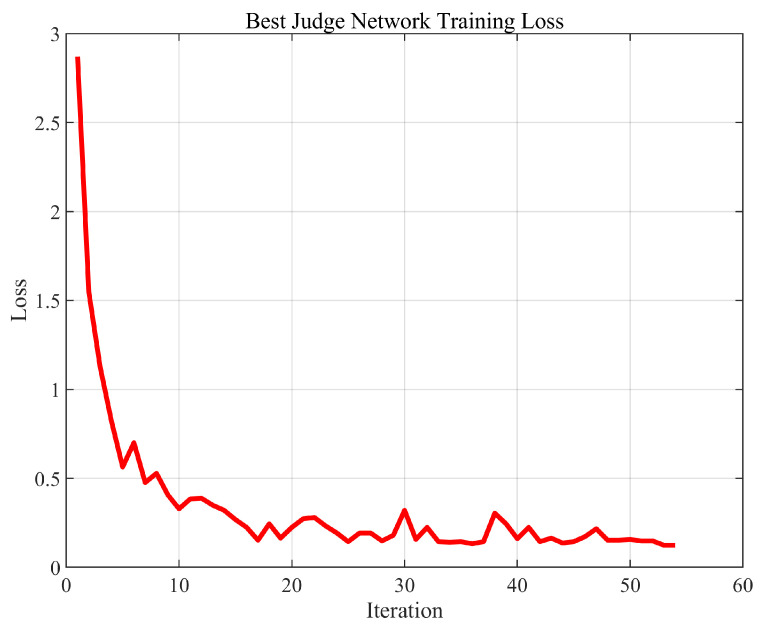
Best base station judgment network training loss.

The performance of the base station judgment network was first evaluated. Figure 5 shows the training loss curve, which gradually converges to near zero with minor oscillations, indicating that the network has reached stable convergence.

**Figure 6:**
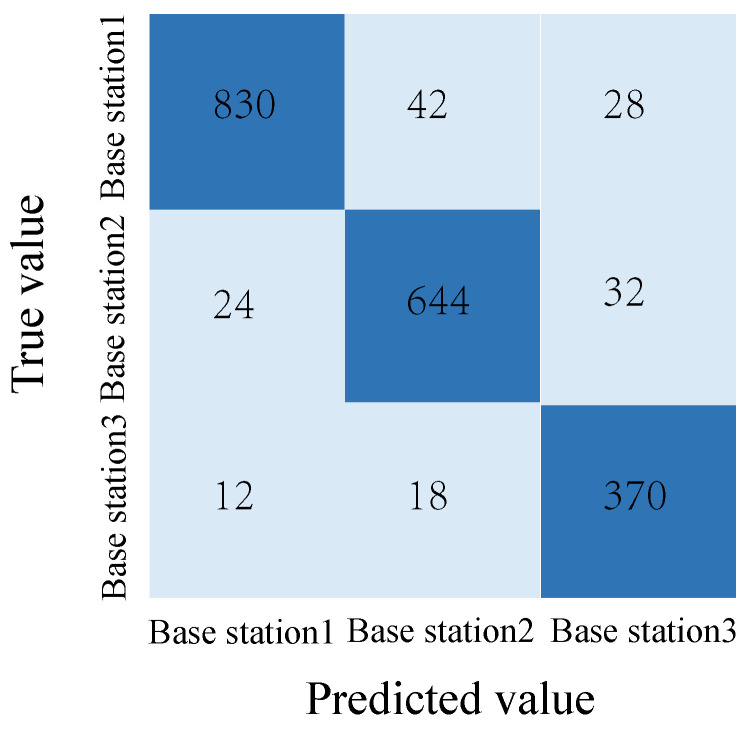
Confusion matrix of base station judgment.

To further analyze the classification performance, the confusion matrix on the test set is shown in Figure 6. The diagonal elements denote accurate classifications for respective base stations, while off-diagonal elements signify prediction errors. Specifically, Base Station 1, 2, and 3 have 830, 644, and 370 correct predictions, respectively.

Furthermore, this paper calculated classification metrics for each base station category, including precision, recall, and F1 score. The precision for the three base stations was 95.84%, 91.48%, and 89.74%, respectively. The recall for the three base stations was 92.22%, 92.00%, and 92.50%, respectively. Their F1 scores were 94.00%, 91.74%, and 91.10%, respectively. These metrics demonstrate that the model’s prediction performance is relatively balanced across base station types, with the highest precision achieved for base station 1, which has the most samples. Although base station 3 has the fewest samples, it still maintains a good recall and F1 score, demonstrating the model’s ability to effectively identify small-category samples.

**Figure 7:**
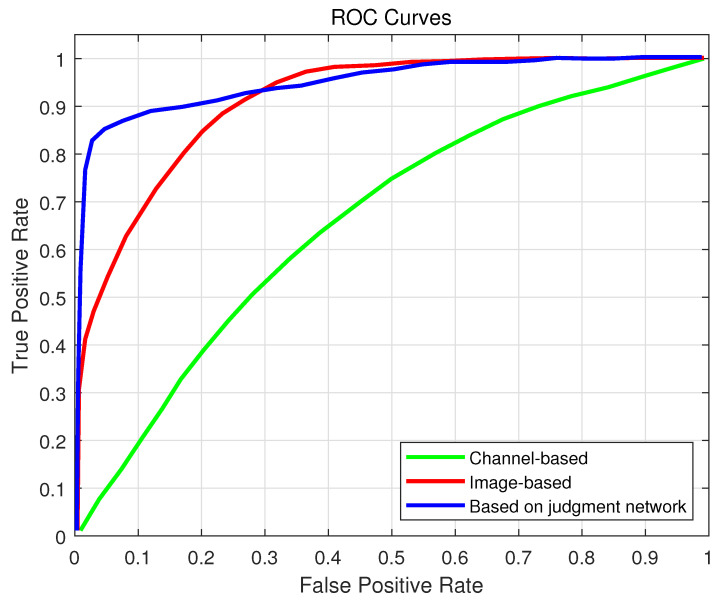
ROC diagram for determining the best base station.

To demonstrate the advantage of multimodal feature extraction, single-modal networks were also evaluated: one based solely on channel matrices and another on image data. The ROC curves are illustrated in Figure 7, where the proposed multimodal network consistently outperforms both single-modal models. This indicates that combining visual scene semantics and channel information significantly enhances the network’s capability to accurately determine the best base station.

As shown in Table 2, the comparative performance results demonstrate the superiority of the proposed MOBS-Net over existing judgment networks. Specifically, MOBS-Net achieves the highest overall accuracy of 92.2%, exceeding other methods by a considerable margin. With per-BS F1 scores maintained in a narrow range of 91.10% to 94.00%, the model attains remarkable stability, reflecting its well-balanced design. exhibits remarkable robustness, achieving 81% accuracy in the presence of image noise and 90% under channel noise. These results validate the adaptive noise-resilient capability of the proposed network design. In addition, MOBS-Net demonstrates an inference speed of only 8.7 ms, which is significantly faster than other compared models while maintaining superior accuracy. Collectively, these results confirm that MOBS-Net achieves an effective balance among accuracy, robustness, and computational efficiency, highlighting its suitability for real-time deployment in integrated communication and perception systems.

#### 4.3.2. Multimodal Base Station Prediction Network

**Figure 8:**
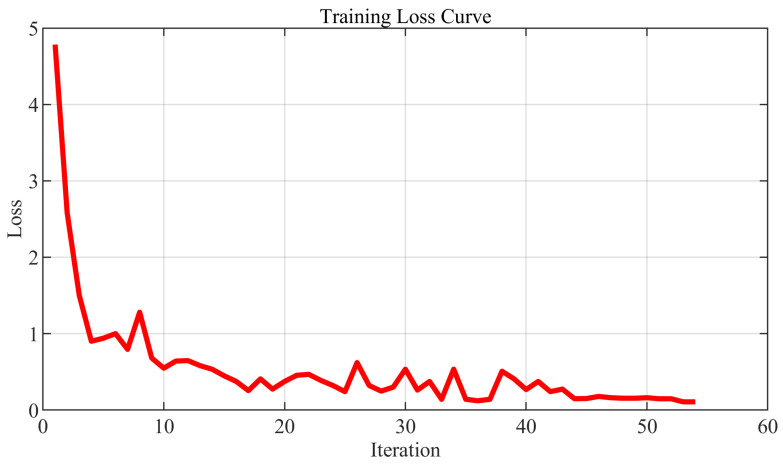
Best base station prediction network training loss.

**Figure 9:**
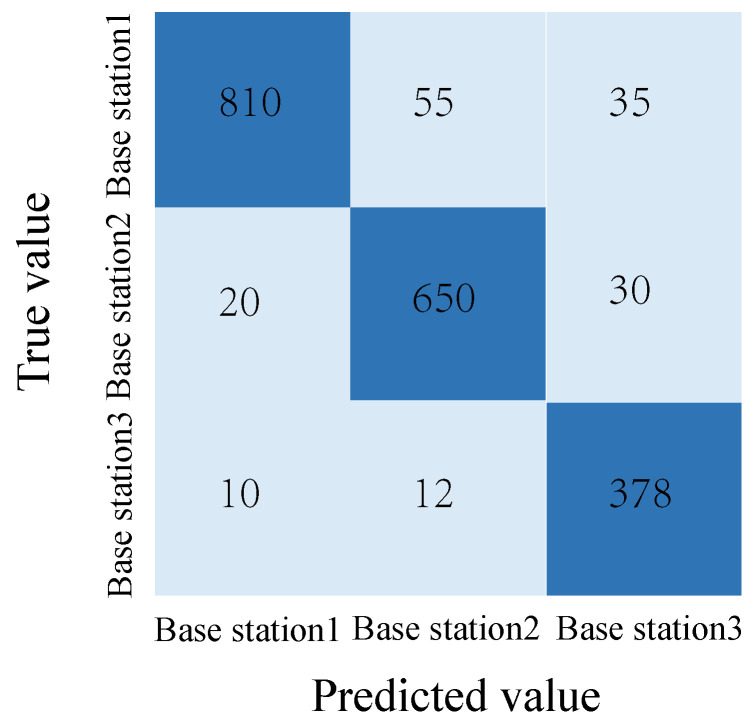
Confusion matrix of the best base station prediction network.

The proposed multimodal base station prediction network was further tested. Figure 8 shows the training loss, which gradually converges, reflecting stable training behavior. Figure 9 displays the confusion matrix for the prediction network. Base Station 1 achieves 810 correct predictions, Base Station 2 reaches 650, and Base Station 3 has 378. Class-wise precision, recall, and F1-scores demonstrate balanced performance: Base Station 1 achieves a precision of 96.43%, recall of 90.00%, and F1-score of 93.10%; Base Station 2 shows a precision of 90.60%, recall of 92.86%, and F1-score of 91.71%; Base Station 3 maintains a precision of 85.37%, recall of 94.50%, and F1-score of 89.69%. These results indicate that the model performs robustly across both majority and minority classes.

**Figure 10:**
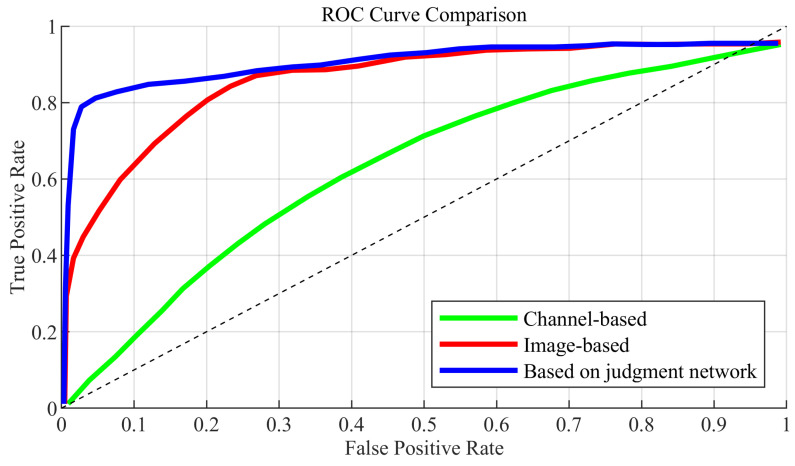
ROC diagram of the best base station prediction network.

To assess the impact of modality selection, two single-modal prediction models were evaluated: one using only partial channel matrix sequences and another using only multi-view image sequences. ROC curves comparing the three networks are shown in Figure 10. Across all false positive rates, the multimodal network maintains higher true positive rates—a performance gain stemming from its synergistic use of multi-modal inputs to better capture spatio-temporal dependencies, ultimately boosting prediction accuracy. Interestingly, the image-only model outperforms the channel-only model, suggesting that visual information is more informative for spatial positioning and obstruction recognition, whereas partial channel matrices may lose relevant temporal and spatial features due to compression or limited representation.

It should be emphasized that Figure 7 and Figure 10 provide an ablation comparison between single-modal and multimodal configurations. Specifically, the image-only, channel-only, and multimodal versions are evaluated for performance attribution across the input modalities. The results confirm that multimodal fusion significantly enhances both classification accuracy and robustness, validating the importance of combining image, channel, and location information in the proposed framework.

**Figure 11:**
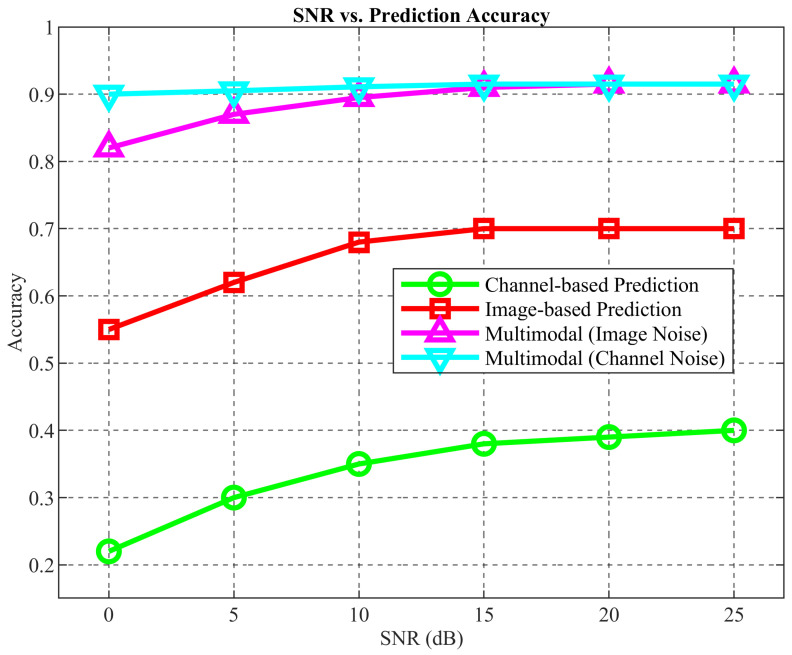
Optimal base station prediction network performance with noise.

The robustness of the proposed multimodal network under noisy environments was evaluated, as shown in Figure 11. Under image noise, the prediction accuracy remains stable and relatively high, even at low SNR, with a minimum accuracy of 81%. Under channel noise, the network achieves up to 90% accuracy, while single-modal models perform worse, with the channel-only model dropping below 40% accuracy under low SNR, and the image-only model decreasing to 56%.These results validate that multimodal fusion effectively counters the detrimental effects of environmental noise, providing a reliable solution for base station prediction in complex communication scenarios.

Table 3 summarizes the performance comparison among different prediction networks. The proposed MOBS-Net demonstrates superior predictive capability, achieving the highest prediction accuracy of 91.5% and a blockage F1 score of 92%. These results indicate that MOBS-Net can effectively capture dynamic spatial–temporal correlations and accurately anticipate potential blockages in communication scenarios. Moreover, the inference time of 12.3 ms shows that the proposed model maintains high computational efficiency, outperforming other approaches such as those in [13,14,16], which require up to 40 ms per inference. The prediction window of 0.4 s (corresponding to four frames) further confirms that MOBS-Net achieves fast and responsive forecasting suitable for real-time adaptive transmission. Overall, these results verify that MOBS-Net effectively balances accuracy, robustness, and latency, demonstrating strong potential for deployment in time-critical wireless perception and prediction systems.

## 5. Conclusions

In this study, we proposed the Multimodal Optimal Base Station Selection Network (MOBS-Net), which integrates a real-time base station judgment network with a sequential base station prediction network. The judgment module leverages a convolutional neural network to extract image features and employs a Transformer-based fusion mechanism to integrate multimodal information, thereby improving the accuracy of optimal base station decisions. Meanwhile, the prediction module exploits multimodal temporal data by introducing a large-scale multimodal model to capture image-sequential semantics and refine task-specific predictions, enhancing the continuity of image understanding and prediction accuracy. Experimental results demonstrate that MOBS-Net significantly outperforms single-modal approaches. Specifically, the optimal base station judgment accuracy reached 92.20%, while the prediction accuracy attained 91.5%. These results confirm the reliability of MOBS-Net in sensing channel states and its capability to proactively switch base stations based on anticipated conditions. It is worth noting that the current evaluation is conducted on a dataset containing three base stations, which may limit the scalability analysis of the proposed network. In future work, we will extend MOBS-Net to large-scale scenarios with dense base station deployments to verify its adaptability and performance in complex 6G environments. Future work will further incorporate recent multimodal transformer-based frameworks and graph-enhanced base station selection methods to provide a more comprehensive performance comparison and validate the scalability of the proposed MOBS-Net. Furthermore, future work will incorporate recent multimodal transformer-based frameworks and graph-enhanced base station selection methods to provide a more comprehensive performance comparison and further validate the scalability of the proposed MOBS-Net.

## Figures and Tables

**Table 1:** Base station parameters.

Base Station	X Coordinate	Y Coordinate	Z Coordinate	Roll	Pitch	Yaw
Base Station 1	−9.7702	−54.9763	0.4	0	0	0
Base Station 2	−8.1531	−87.2525	0.4	0	0	0
Base Station 3	20.7087	−87.1356	0.4	0	0	90

**Table 2:** Performance Comparison of Judgment Networks.

Method	Accuracy	Per-BS F1 Score	Noise Robustness (0 dB SNR)	Inference Time
MOBS-Net (Ours)	92.2%	91.10–94.00% (BS 1–3)	Image Noise: 81% Channel Noise: 90%	8.7 ms
[17]	91.5%	87.75–92.88% (BS 1–4)	Image Noise: 78%	15.2 ms
[15]	75%	-	-	10.3 ms
[16]	82.3%	79.5–85.1%	Image Noise: 65%	9.1 ms

**Table 3:** Performance Comparison of Prediction Networks.

Method	Prediction Accuracy	Blockage F1 Score	Inference Time	Prediction Window
MOBS-Net (Ours)	91.5%	92%	12.3 ms	0.4 s (4 frames)
[14]	88%	0.87	22.5 ms	0.5 s
[13]	86.5%	-	40 ms	0.3 s
[16]	79.2%	0.78	18.6 ms	0.4 s

## Data Availability

The original contributions presented in this study are included in the article. Further inquiries can be directed to the corresponding author.

## References

[1] Chen M., Liu M., Wang W., Dou H., Wang L. (2023). Cross-modal semantic communications in 6g. Proceedings of the 2023 IEEE/CIC International Conference on Communications in China (ICCC).

[2] Cao Y., Li A., Lou J., Chen M., Zhang X., Kang B. (2021). An attention-based bidirectional gated recurrent unit network for location prediction. Proceedings of the 2021 13th International Conference on Wireless Communications and Signal Processing (WCSP).

[3] Rong Y., Mao Y., He X., Chen M. (2025). Large-scale traffic flow forecast with lightweight llm in edge intelligence. IEEE Internet Things Mag..

[4] Haider M., Ahmed I., Rubaai A., Pu C., Rawat D.B. (2023). Gan-based channel estimation for irs-aided communication systems. IEEE Trans. Veh. Technol..

[5] Rong Y., Mao Y., Cui H., He X., Chen M. (2025). Edge computing enabled large-scale traffic flow prediction with gpt in intelligent autonomous transport system for 6g network. IEEE Trans. Intell. Transp. Syst..

[6] Mingkai C., Minghao L., Zhe Z., Zhiping X., Lei W. (2024). Task-oriented semantic communication with foundation models. China Commun..

[7] Chen M., Liu M., Wang C., Song X., Zhang Z., Xie Y., Wang L. (2024). Cross-modal graph semantic communication assisted by generative ai in the metaverse for 6g. Research.

[8] Chen M., Zhao L., Chen J., Wei X., Guizani M. (2023). Modal-aware resource allocation for cross-modal collaborative communication in iiot. IEEE Internet Things J..

[9] Wang L., Yin A., Jiang X., Chen M., Dev K., Qureshi N.M.F., Yao J., Zheng B. (2022). Resource allocation for multi-traffic in cross-modal communications. IEEE Trans. Netw. Serv. Manag..

[10] Chen M., Wei X., Chen J., Wang L., Zhou L. (2020). Integration and provision for city public service in smart city cloud union: Architecture and analysis. IEEE Wirel. Commun..

[11] Peng Q., Li J., Shi H. (2022). Deep learning based channel estimation for ofdm systems with doubly selective channel. IEEE Commun. Lett..

[12] Kim W., Ahn Y., Kim J., Shim B. (2023). Towards deep learning-aided wireless channel estimation and channel state information feedback for 6g. J. Commun. Netw..

[13] Charan G., Alrabeiah M., Alkhateeb A. (2021). Vision-aided 6g wireless communications: Blockage prediction and proactive handoff. IEEE Trans. Veh. Technol..

[14] Charan G., Alrabeiah M., Alkhateeb A. (2021). Vision-aided dynamic blockage prediction for 6g wireless communication networks. Proceedings of the 2021 IEEE International Conference on Communications Workshops (ICC Workshops).

[15] Charan G., Osman T., Hredzak A., Thawdar N., Alkhateeb A. (2022). Vision-position multi-modal beam prediction using real millimeter wave datasets. Proceedings of the 2022 IEEE Wireless Communications and Networking Conference (WCNC).

[16] Charan G., Alkhateeb A. (2022). Computer vision aided blockage prediction in real-world millimeter wave deployments. Proceedings of the 2022 IEEE Globecom Workshops (GC Wkshps).

[17] Lin B., Gao F., Zhang Y., Pan C., Liu G. (2022). Multi-camera view based proactive bs selection and beam switching for v2x. arXiv.

[18] Soltani M., Pourahmadi V., Sheikhzadeh H. (2020). Pilot pattern design for deep learning-based channel estimation in ofdm systems. IEEE Wirel. Commun. Lett..

[19] Bai Q., Wang J., Zhang Y., Song J. (2019). Deep learning-based channel estimation algorithm over time selective fading channels. IEEE Trans. Cogn. Commun. Netw..

[20] Mei K., Liu J., Zhang X., Rajatheva N., Wei J. (2021). Performance analysis on machine learning-based channel estimation. IEEE Trans. Commun..

[21] Zhang H., Zhang L., Jiang Y., Wu Z. (2021). Lstm and resnets deep learning aided end-to-end intelligent communication systems. Proceedings of the 2021 2nd Information Communication Technologies Conference (ICTC).

[22] Liu Y., Al-Nahhal I., Dobre O.A., Wang F. (2022). Deep-learning channel estimation for irs-assisted integrated sensing and communication system. IEEE Trans. Veh. Technol..

[23] Zhang Z., Ji T., Shi H., Li C., Huang Y., Yang L. (2023). A self-supervised learning-based channel estimation for irs-aided communication without ground truth. IEEE Trans. Wirel. Commun..

[24] Yi X., Zhong C. (2020). Deep learning for joint channel estimation and signal detection in ofdm systems. IEEE Commun. Lett..

[25] Zhang S., Liu Y., Gao F., Xing C., An J., Dobre O.A. (2021). Deep learning based channel extrapolation for large-scale antenna systems: Opportunities, challenges and solutions. IEEE Wirel. Commun..

[26] Chen M., Gu Y., He X., Wang S., Huang F., Wang L. (2025). Explainable artificial intelligence enhance image semantic communication system in 6g-iot. IEEE Internet Things J..

[27] Ye M., Pan C., Xu Y., Li C. (2024). Generative adversarial networks-based channel estimation for intelligent reflecting surface assisted mmwave mimo systems. IEEE Trans. Cogn. Commun. Netw..

[28] Singh S., Trivedi A., Saxena D. (2024). Generative channel estimation for intelligent reflecting surface-aided wireless communication. Wirel. Netw..

[29] Singh J., Singh K., Janu D., Kumar S., Singh G. (2025). Deep learning-driven channel estimation for intelligent reflecting surfaces aided networks: A comprehensive survey. Eng. Appl. Artif. Intell..

[30] Safari M.S., Pourahmadi V., Sodagari S. (2019). Deep ul2dl: Data-driven channel knowledge transfer from uplink to downlink. IEEE Open J. Veh. Technol..

[31] Yang Y., Zhang S., Gao F., Xu C., Ma J., Dobre O.A. (2020). Deep learning based antenna selection for channel extrapolation in fdd massive mimo. Proceedings of the 2020 International Conference on Wireless Communications and Signal Processing (WCSP).

[32] Liu C., Liu X., Ng D.W.K., Yuan J. (2021). Deep residual learning for channel estimation in intelligent reflecting surface-assisted multi-user communications. IEEE Trans. Wirel. Commun..

[33] Dosovitskiy A. (2020). An image is worth 16 × 16 words: Transformers for image recognition at scale. arXiv.

[34] Lu J., Batra D., Parikh D., Lee S. (2019). Vilbert: Pretraining task-agnostic visiolinguistic representations for vision-and-language tasks. Adv. Neural Inf. Process. Syst..

[35] Radford A., Kim J.W., Hallacy C., Ramesh A., Goh G., Agarwal S., Sastry G., Askell A., Mishkin P., Clark J. (2021). Learning transferable visual models from natural language supervision. Proceedings of the International Conference on Machine Learning.

[36] Li J., Li D., Savarese S., Hoi S. (2023). Blip-2: Bootstrapping language-image pre-training with frozen image encoders and large language models. Proceedings of the International Conference on Machine Learning.

[37] Cheng X., Huang Z., Bai L., Zhang H., Sun M., Liu B., Li S., Zhang J., Lee M. (2023). M^3^SC: A generic dataset for mixed multi-modal (mmm) sensing and communication integration. China Commun..

